# Behavioral Changes Over Time Following Ayahuasca Exposure in Zebrafish

**DOI:** 10.3389/fnbeh.2017.00139

**Published:** 2017-07-28

**Authors:** Robson Savoldi, Daniel Polari, Jaquelinne Pinheiro-da-Silva, Priscila F. Silva, Bruno Lobao-Soares, Mauricio Yonamine, Fulvio A. M. Freire, Ana C. Luchiari

**Affiliations:** ^1^Luchiari Lab, Physiology, Federal University of Rio Grande do Norte Natal, Brazil; ^2^Biophysics and Pharmacology, Federal University of Rio Grande do Norte Natal, Brazil; ^3^Clinical and Toxicological Analysis, University of São Paulo São Paulo, Brazil; ^4^Aquatic Fauna Lab, Botany and Zoology, Federal University of Rio Grande do Norte Natal, Brazil

**Keywords:** entheogen, psychoactive, drug, medicine, anxiety-like behavior, depression

## Abstract

The combined infusion of *Banisteriopsis caapi* stem and *Psychotria viridis* leaves, known as ayahuasca, has been used for centuries by indigenous tribes. The infusion is rich in *N*, *N*-dimethyltryptamine (DMT) and monoamine oxidase inhibitors, with properties similar to those of serotonin. Despite substantial progress in the development of new drugs to treat anxiety and depression, current treatments have several limitations. Alternative drugs, such as ayahuasca, may shed light on these disorders. Here, we present time-course behavioral changes induced by ayahuasca in zebrafish, as first step toward establishing an ideal concentration for pre-clinical evaluations. We exposed adult zebrafish to five concentrations of the ayahuasca infusion: 0 (control), 0.1, 0.5, 1, and 3 ml/L (*n* = 14 each group), and behavior was recorded for 60 min. We evaluated swimming speed, distance traveled, freezing and bottom dwelling every min for 60 min. Swimming speed and distance traveled decreased with an increase in ayahuasca concentration while freezing increased with 1 and 3 ml/L. Bottom dwelling increased with 1 and 3 ml/L, but declined with 0.1 ml/L. Our data suggest that small amounts of ayahuasca do not affect locomotion and reduce anxiety-like behavior in zebrafish, while increased doses of the drug lead to crescent anxiogenic effects. We conclude that the temporal analysis of zebrafish behavior is a sensitive method for the study of ayahuasca-induced functional changes in the vertebrate brain.

## Introduction

The ayahuasca brew has been used for centuries for religious and medicinal purposes by a number of groups in South America, notably the indigenous people from the Amazon region (McKenna et al., 1984). Ayahuasca is traditionally made from the decoction of *Banisteriopsis caapi* stalks and *Psychotria viridis* leaves.

*Banisteriopsis caapi* contains the alkaloids harmine, tetrahydroharmine (THH) and, in smaller amounts, harmaline, all belonging to the β-carboline group, in addition to monoamine oxidase inhibitors (MAOIs). *P. viridis* is rich in *N*, *N*-dimethyltryptamine (DMT) (McKenna et al., 1984; Riba et al., 2003). After ingestion, DMT is inactivated by MAO found in the intestines and the liver, but in the presence of MAOI (from *B. caapi*), DMT remains active and plays a role in the central nervous system (Ray, 2010). In addition to the protective effect of DMT, the inhibition of MAO itself modulates the viability of monoaminergic neurotransmitters (serotonin, noradrenaline, and dopamine), increasing its bioavailability (McKenna et al., 1984; Riba et al., 2003). Functionally, DMT’s properties are similar to those of serotonin and other tryptamines, such as psilocybin, and act essentially as agonists on 5-HT2A and 5-HT2C receptors (Ray, 2010).

It is the synergistic interaction of the two plants that make up the ayahuasca brew that confers its complex effects, which include sedation, ideation, space-time scale changes, sharpening of mental images, increased sound perception, dissociative perception, feelings of well-being, and increased interception (Shanon, 2002; Riba et al., 2003; de Araujo et al., 2012). In humans, the most common effects include sensory, cognitive and affective alterations (Almeida Prado et al., 2009), complex visual occurrences (de Araujo et al., 2012), and even entheogenic experiences (Shanon, 2003). The effects start from 35 to 40 min after ingesting the infusion, reach maximum intensity between 90 and 120 min, and end around 4 h after administration (Riba et al., 2003). It has been shown that the ritualistic use of ayahuasca is not associated with psychosocial problems commonly observed for drugs of abuse (Fábregas et al., 2010). For instance, dos Santos et al. (2007) observed a significant reduction in the panic and hopelessness behavior rating scale in individuals under the effect of the substance. There are suggestions for the use of ayahuasca as a modulating agent for anxiety, panic, and associated disorders (Riba et al., 2002; dos Santos et al., 2015, 2016a,b).

On one hand, studies have indicated safety and tolerability with the use of ayahuasca, mainly based on reports from individuals using it for more than 30 years with no evidence of impaired health (Grob et al., 1996; Callaway et al., 1999; Riba et al., 2001, 2003). Furthermore, there is no evidence of any effects of ayahuasca on physiological parameters such as blood pressure (systolic and diastolic) and heart rate (Riba et al., 2003). However, studies on acute toxicity and toxicity promoted by the prolonged and repeated use of different concentrations are lacking (Gable, 2007).

On the other hand, the pharmacology of ayahuasca brew substances leads one to believe in its potential for reducing symptoms of anxiety and depression, and possibly exhibiting advantages over currently used drugs. Anxiety and depression disorders are a combination of physiological, psychological, and environmental factors (Rutter, 2003; Venzala et al., 2013; Adhikari, 2014). According to the National Collaborating Centre for Mental Health (2010), these disorders are the most common mental health problems in our society, displaying a series of emotional and functional challenges. Despite substantial progress in developing new drugs and treatments, less than 50% of patients achieve remission after treatment with conventional antidepressants, and even after combined treatments with up to 4 systematically applied drugs, about one-third fail to achieve remission (Warden et al., 2007). In addition to this low effectiveness, current pharmacological treatments appear to have limitations associated with the time course of the drug, which takes several weeks to achieve the desired therapeutic effects (Zarate et al., 2006; Zarate, 2011).

At present, the zebrafish is one of the most promising models for studies on stress, anxiety, and related disorders (Egan et al., 2009). This tiny fish is widely used in neuroethological research and has been suggested for behavioral screening of drugs due to the ideal balance between the complexity of its physiological system and the simplicity of its biological model. Zebrafish share a considerable number of molecular pathways, proteins and protein subproducts and exhibit 70–80% genome homology with humans (Holzschuh et al., 2001; Kaslin et al., 2004; Crollius and Weissenbach, 2005; Faraco et al., 2006; Prober et al., 2006). The zebrafish is not only an ideal model for behavioral screening, but most of the genes identified in this species are conserved and have homologs in mammals (Cerdà et al., 1998; Crollius and Weissenbach, 2005), enabling the examination of brain function and the development of brain diseases (Kalueff et al., 2014). The zebrafish is an important model for research on psychoactive substances because its brain structure (Kolb and Whishaw, 1998; Klee et al., 2012) and neurochemistry (Chatterjee and Gerlai, 2009; Gerlai et al., 2009) offer translational relevance to humans (Crollius and Weissenbach, 2005) and allow the model to be explored for a thorough understanding of the effects of substances used/abused by humans. In fact, several studies have shown that zebrafish respond similarly to mammals when treated with many pharmacological compounds (Stewart et al., 2014; Vacaru et al., 2014; Abreu et al., 2016). As such, we used the zebrafish to determine the effect of different concentrations of ayahuasca brew on behavior, an important screening tool for the development of methodologies to assess the effect of the substance on physiology and cognition. Our dose-response analysis over time is the first study of the effects of ayahuasca on zebrafish.

## Materials and Methods

### Stock and Housing

Adult zebrafish (wild-type, both sexes) were acquired from a local breeding farm (Natal, Brazil) and held in high-density system tanks in the vivarium of the Fish Laboratory [Physiology Department, Federal University of Rio Grande (UFRN)]. The vivarium contained four 50 L-tanks that formed a recirculation system with multistage filtration including a mechanical filter, biological filter, activated carbon filter, and a UV light sterilizing unit. Ambient and water temperature were kept at 28°C, and pH at 7.0. The photoperiod was set at 12L:12D (12 h light:12 h dark), with light intensity of 250 lx. Fish were fed twice daily with brine shrimp and commercial diet. Experimental procedures were approved by the Animal Ethics Committee of the Federal University of Rio Grande do Norte (CEUA 053/2016).

### Ayahuasca Infusion Preparation

The ayahuasca brew was donated by the ‘Igreja da Barquinha’ based in the city of Ji-Paraná, Rondonia state, Brazil. The following traditional recipe was used: 50% *B. caapi* stalks and 50% *P. viridis* leaves added to water and boiled to concentration for several hours. The infused water was transferred to plastic bottles and stored in a refrigerator. The infusion used in the present study came from the original batch.

The quantification of alkaloids in the batch was evaluated by the Department of Clinical Analysis and Toxicology (USP) by mass spectroscopy, according to the procedure described in Pires et al. (2009). From the original solution, 50 μL of sample was diluted with deionized water (1:100) and 500 μL of the diluted sample solution was added with 3 ml of 0.25 M Borate buffer, pH 9.0 and the internal standard diphenhydramine (100 μL of a solution of 10 μg/mL) was loaded onto a previously conditioned (2.0 mL methanol, 1.0 mL deionized water, and 2.0 mL borate buffer) C18 cartridge (Classic Sep-Pack^®^C18 cartridges, 360 mg from Waters, Co., Bellefonte, PA, United States). The cartridge was washed with 1.0 ml deionized water and 1.0 ml of 10% acetonitrile, left to dry for 7 min, and the analytes were eluted with 2.0 ml methanol. Next, 2 μL of the solution were injected into the GC-NPD system. Analyses of *N*, *N*-Dimethyltryptamine, harmine, harmaline, and tetrahydroharmine were conducted using an Agilent 6890 gas chromatograph equipped with a nitrogen–phosphorous detector and 7683 series automatic injector (Little Falls, DE, United States). Chromatographic separation was performed on an HP Ultra-2 fused-silica capillary column (25 m × 0.2 mm × 0.33 μm film thickness) using ultra-pure-grade nitrogen as carrier gas at 1.0 mL/min in a constant flow rate mode. Injections of 2 μL were made in splitless mode. The injector port and detector temperature was 280°C. The oven was kept at 70°C for 1 min; programmed at 30°C/min to 120°C and 20°C/min to 300°C with a hold at 300°C for 4 min. The analytes were identified by comparing their relative retention time with the corresponding values of the internal standard diphenhydramine assayed in the same run. The amount of the products was estimated by the ratio of the integrated peak area to the internal standard. Every result was multiplied by 100 to compensate for initial dilution. The ayahuasca used in this study contained (mean ± SD) 0.36 ± 0.01 mg/mL of DMT, 1.86 ± 0.11 mg/mL of harmine, 0.24 ± 0.03 mg/mL of harmaline, and 1.20 ± 0.05 mg/mL of tetrahydroharmine. de Castro-Neto et al. (2013) used a similar composition of ayahuasca with 0.59 mg/mL of DMT, 5.09 mg/mL of harmine, 0.19 mg/mL of harmaline, and 0.99 mg/mL of tetrahydroharmine. According to these authors, the Brazilian ayahuasca is composed, in average, of 0.6 mg/mL of DMT, 1.2 mg/mL of harmine, 0.2 mg/mL of harmaline and 1.07 mg/mL of tetrahydroharmine, comparable to the proportions found in our brew. Tetrahydroharmine was synthesized according to the procedure described in Callaway et al. (1996).

### Ayahuasca Exposure

To determine the time-course effect of ayahuasca doses on zebrafish, 70 animals (3.42 ± 0.85 g) were randomly assigned to different experimental groups corresponding to each ayahuasca concentration (*n* = 14 in each group). This experimental design used five acute challenge concentrations: 0.0 (control), 0.1, 0.5, 1.0, 3.0 ml/L of ayahuasca.

Fish were initially held in groups of 14 in glass tanks (50 cm × 30 cm × 25 cm, width × depth × height; 37 L) for 7 days to acclimatize them to the test room. The bottom and back of the holding tanks were covered with white paper to provide a uniform environment. During this period, water quality was the same as in the stock condition, with filtration and oxygen renewal provided by 140 Bio Wheel power filters. Food was offered twice daily.

Smaller tanks (15 cm × 15 cm × 10 cm, 25 L) were used for the behavioral assay. Ayahuasca was added directly to the test water to achieve each test concentration. Fish were individually transferred to the test tanks and behavior was recorded for 60 min using an HD camcorder (Sony DCR-SX45 Digital Video Camera Recorder) placed 1 m in front of the tanks. Sixty min exposure was chosen due to the peak of ayahuasca effects occurring at 1 h from the administration and decreasing to completely disappear after 6–8 h, as described by Riba et al. (2002).

Fish behavioral records were tracked using Zebtrack software developed in MatLab (Pinheiro-da-Silva et al., 2017). The behavioral variables measured were average swimming speed, total distance traveled, duration of immobility (freezing) and bottom dwelling (distance from the bottom of the tank). Average swimming speed and total distance traveled during the 60-min period refer to fish activity, increased swimming speed and distance traveled are usually related to stress and avoidance (fear, anxiety), while decreased speed and distance traveled are associated with depressive behavior (Kalueff et al., 2013). The other two parameters, freezing and distance from the bottom of the tank (also called diving or geotaxis), are related to anxiety-like behavior (Maximino et al., 2010, 2014; Kalueff et al., 2013). Increased freezing is a result of anxiety/stress, while a short distance from the bottom is usually a highly sensitive indicator of anxiety (Kalueff et al., 2013).

### Statistical Analysis

Data were initially evaluated for potential problems with outliers, homogeneity, normality, zero trouble, collinearity and variable independence, as suggested by Zuur et al. (2010). Next, mixed-effects model analysis for longitudinal data was carried out to develop a model for the response variable (recorded behavioral parameters) and the explanatory variable (60-min time course and ayahuasca concentrations). The longitudinal aspect of the data considered its repeated measures characteristic (over time) (Zuur et al., 2010). The mixed model showed random effect factors (represented by behavioral variation within each group), fixed effect factors (represented by the effect of explicative variables: concentration) and error.

To construct the mixed model, we used the glmmPQL command from the MASS package (Venables and Ripley, 2003) of the R program (R Development Core Team, 2015). The algorithm on the glmmPQL command was applied due to the abnormal distribution and dispersion exhibited by the residuals of the response variable during exploratory analysis. The response variables freezing and bottom dwelling were discrete quantitative data that varied between 0 and 60 s (freezing) and 0 and 10 cm (bottom distance), which displayed a binomial distribution error with logit link function (according to Zuur et al., 2010). Since the response variables average speed and distance traveled were positive continuous quantitative data, not including zero (Y > 0), a goodness-of-fit test determined that the best distribution function for these data sets was the gamma function (link function = inverse). In all cases, the *post hoc* comparisons between treatments of each model were made using the Tukey *post hoc* test (lsmeans package) (Lenth and Hervé, 2014).

The behavioral parameters evaluated were also compared in terms of the area under the curve (AUC). The behavioral variables (average speed, total distance traveled, freezing and bottom dwelling) and the ayahuasca concentrations tested in this study were used to calculate AUC through the Bolstad package (Curran, 2013), which perform the integral calculations using Simpson’s Rule. After that, AUCs for each behavior parameter were tested using one-way ANOVA followed by Tukey *post hoc* test through Multicomp package (Hothorn et al., 2008). For all comparisons, the significance level was set at *p* < 0.05.

## Results

**FIGURE 1:**
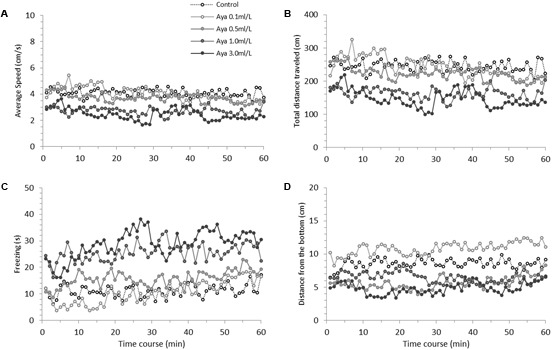
Time-course behavioral changes during 60-min Ayahuasca exposure in zebrafish. **(A)** Average swimming speed, **(B)** Total distance traveled, **(C)** Freezing and **(D)** Distance from the bottom of the tank. **(A,B)** Indicate locomotion parameters, **(C,D)** indicate anxiety-like behavior parameters. The Ayahuasca doses are shown in above graph **(A)**. Sample sizes (n) were 14 for each dose. For statistical analysis see Results and **Table 1**.

**FIGURE 2:**
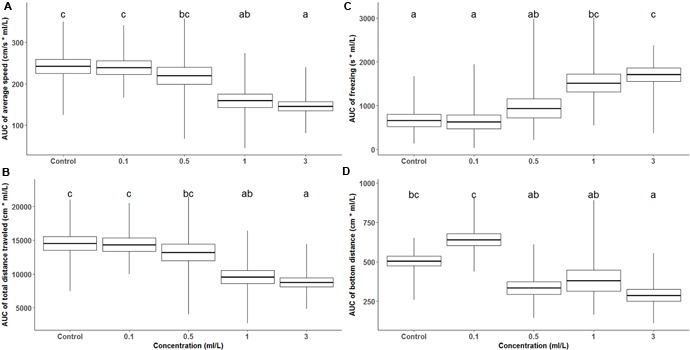
Box plot shows average and SEM of the area under the curve (AUC) for the locomotor behavior of zebrafish during 60-min Ayahuasca exposure: **(A)** average swimming speed, **(B)** total distance traveled, **(C)** freezing, and **(D)** distance from the bottom of the tank, whiskers represent the range. In each graph, at least one different letter indicates statistical difference by one-way ANOVA (*p* < 0.05).

**Figure 1** presents zebrafish behavioral parameters measured during a 60-min recording session under the effects of acutely administered ayahuasca. **Figure 2** depicts the box plots representing the average AUC of behavioral parameters evaluated for fish exposed to ayahuasca for 60 min.

Mixed model comparison showed that average speed changed over time (GLMM, χ^2^ = 8.41, df = 1, *p* < 0.004; **Table 1**) and due to the ayahuasca concentration used (GLMM, χ^2^ = 19.91, df = 4, *p* = 0.0005; **Table 1**). The *post hoc* comparison test (lsmeans) between ayahuasca concentrations indicated that 0.0 (control) and 0.1 ml/L of ayahuasca differed from 1.0 and 3.0 ml/L in average speed, suggesting that a high concentration causes a decline in swimming speed compared to a low concentration (0.1 ml/L). The same pattern was observed for total distance traveled; mixed model comparison indicated changes over time (GLMM, χ^2^ = 8.44, df = 1, *p* = 0.003; **Table 1**) according to the concentration used (GLMM, χ^2^ = 19.93, df = 4, *p* = 0.0005; **Table 1**). Lsmeans comparison between ayahuasca concentrations also exhibited statistical differences between low (0.0 and 0.1 ml/L) and high concentrations (1.0 and 3.0 ml/L), with high concentrations decreasing distance traveled compared to low concentrations.

**Table 1:** Estimates of mixed effect model for the behavioral parameters measured during Ayahuasca exposure.

						Behavioral parameters
	**Average speed**			**Total distance traveled**			**Freezing**			**Distance from bottom**		
**Explanatory variable**	**Chi-squared**	***p*-Value**		**Chi-squared**	***p*-Value**		**Chi-squared**	***p*-Value**		**Chi-squared**	***p*-Value**	

60 min time course	8.41	0.004		8.44	0.003								
Doses	19.91	0.0005		19.93	0.0005								

**Pairwise comparison**	**lsmeans ± SEM**	***t*-Value**	***p*-Value**	**lsmeans ± SEM**	***t*-Value**	***p*-Value**	**lsmeans ± SEM**	***t*-Value**	***p*-Value**	**lsmeans ± SEM**	***t*-Value**	***p*-Value**

Dose 0.0 vs. dose 0.1	0.03 ± 0.05	0.61	0.97	5.31 ± 0.00	0.61	0.99	0.03 ± 3.60	0.01	1.00	-2.34 ± 0.81	-2.86	0.04
Dose 0.0 vs. dose 0.5	-0.04 ± 0.05	-0.84	0.91	-7.14 ± 0.00	-0.83	0.92	-4.61 ± 4.25	-1.08	0.81	2.87 ± 0.81	3.54	0.00
Dose 0.0 vs. dose 1.0	-0.15 ± 0.05	-2.88	0.04	-2.47 ± 0.00	-2.88	0.05	-14.4 ± 4.01	-3.58	0.00	2.10 ± 1.18	1.77	0.03
Dose 0.0 vs. dose 3.0	-0.14 ± 0.05	-2.77	0.03	-2.37 ± 0.00	-2.77	0.04	-17.6 ± 3.44	-5.11	< 0.00	3.71 ± 0.76	4.86	0.00
Dose 0.1 vs. dose 0.5	-0.07 ± 0.05	-1.43	0.60	-1.24 ± 0.00	-1.44	0.61	-4.64 ± 4.48	-1.03	0.83	5.21 ± 0.91	5.69	<0.00
Dose 0.1 vs. dose 1.0	-0.18 ± 0.05	-3.44	0.01	-3.00 ± 0.00	-3.42	0.01	-14.4 ± 4.26	-3.38	0.01	4.44 ± 1.25	3.53	0.00
Dose 0.1 vs. dose 3.0	-0.17 ± 0.05	-3.33	0.01	-2.90 ± 0.00	-3.33	0.01	-17.6 ± 3.73	-4.73	0.00	6.05 ± 0.87	6.92	<0.00
Dose 0.5 vs. dose 1.0	-0.10 ± 0.05	-2.04	0.25	-1.76 ± 0.00	-2.04	0.26	-9.79 ± 4.82	-2.03	0.26	-0.77 ± 1.25	-0.61	0.97
Dose 0.5 vs. dose 3.0	-0.10 ± 0.05	-1.93	0.31	-1.66 ± 0.00	-1.93	0.32	-13.0 ± 4.36	-2.98	0.03	0.84 ± 0.86	0.96	0.86
Dose 1.0 vs. dose 3.0	0.005 ± 0.05	0.11	1.00	9.69 ± 0.00	0.11	0.99	-3.24 ± 4.13	-0.78	0.93	1.61 ± 1.22	1.31	0.68

*Dose values are expressed in mg/L*.

With respect to freezing behavior, mixed model comparison showed that behavior changed over time (GLMM, χ^2^ = 18.53, df = 1, *p* < 0.001; **Table 1**) and in line with the ayahuasca concentration used (GLMM, χ^2^ = 37.74, df = 4, *p* < 0.001; **Table 1**). The *post hoc* comparison test (lsmeans) between ayahuasca concentrations indicated that concentrations higher than 1 ml/L increased freezing behavior compared to low concentrations (0.0 and 0.1 ml/L). For the freezing and bottom dwelling parameters, mixed model comparison showed no changes over time (GLMM, χ^2^ = 0.75, df = 1, *p* = 0.38; **Table 1**), but did exhibit alteration due to exposure to the drug (GLMM, χ^2^ = 44.67, df = 4, *p* < 0.001; **Table 1**). The *post hoc* comparison test (lsmeans) between concentrations demonstrated that those above 0.5 ml/L provoked increased bottom dwelling. The control concentration (0.0 ml/L) also differed from the low concentration of 0.1 mg/L, suggesting that it reduced the distance from the bottom in zebrafish.

**Figure 2A** depicts the box plot of AUC of average speed during the 60 min of ayahuasca exposure, and one-way ANOVA between Ayahuasca concentration indicates that 1 and 3 ml/L concentration differed from control and 0.1 ml/L Ayahuasca concentration (*F*_4,66_ = 7.54, *p* < 0.01). The same pattern was observed for the AUC of total distance traveled (one-way ANOVA, *F*_4,66_ = 7.54, *p* < 0.01), as shown in **Figure 2B**. The anxiety-like behaviors, represented by freezing and distance from the bottom are shown in **Figures 2C,D**. One-way ANOVA indicates high freezing response for high ayahuasca concentrations (*F*_4,66_ = 7.78, *p* < 0.01). **Figure 2D** presents zebrafish distance from the bottom of the test tank (bottom dwelling) and one-way ANOVA showed decreased distance from the bottom for the higher Ayahuasca concentrations (*F*_4,66_ = 10.27, *p* < 0.01).

## Discussion

In the present study, we observed that ayahuasca affects both locomotor and anxiety-like behaviors in zebrafish (*Danio rerio*). While motor behaviors were changed only by high concentrations of the drug, anxiety related behaviors were altered by both high and low concentrations, causing anxiogenic and anxiolytic effects, respectively. This pioneering study on the effects of ayahuasca in zebrafish using a time-course effect showed that ayahuasca exerts a biphasic effect: decreasing anxiety at low levels, and reducing locomotion and increasing anxiety at higher concentrations.

The active components of the ayahuasca brew are DMT (dimethyltryptamine) and MAOI alkaloids harmine, harmaline, and tetrahydroharmine (McKenna et al., 1984). Dimetiltryptamine is a serotonin analog frequently used in combination with the MAOI alkaloids. These elements are known to stimulate the central nervous system, but it is important to underscore that ayahuasca evokes different responses depending on the amount used. Some studies extol the benefits of ayahuasca, suggesting that a single dose may mitigate or even abolish symptoms of anxiety and depression (McKenna, 2004; Barbosa et al., 2005; Dominguéz-Clavé et al., 2016) and users that do not exhibit any health disorders may safely use the brew (Bouso et al., 2013). While the consumption of ayahuasca is rapidly expanding worldwide (Labate and Feeney, 2012; Lanaro et al., 2015), care should be taken due to potential toxic effects reported in several fatal cases from the abuse of DMT (Brush et al., 2004; Muller, 2004; Sklerov et al., 2005; Smolinske et al., 2005; Tanaka et al., 2006; Björnstad et al., 2009; Hill and Thomas, 2011; Fuse-Nagase and Nishikawa, 2013).

In this study, zebrafish exposed to 1 and 3 ml/L of ayahuasca showed decreased swimming speed and distance traveled compared to the other concentrations. Moreover, marked hypoactivity was observed during the entire 60-min period, which was amplified by the end of the observation time (**Figure 1** and **Table 1**). Decreased locomotion and slow swimming for an extended period of time are usually associated with neuromotor deficits, which is the loss of normal neural and/or motor system functioning (Hortopan et al., 2010; Norton and Bally-Cuif, 2010; Hortopan and Baraban, 2011). In zebrafish, hypoactivity is usually attributed to neurotoxicity (Parng et al., 2007; Egan et al., 2009; Kalueff et al., 2013; He et al., 2014). Figueroa (2012) and Pic-Taylor et al. (2015) reported the neurotoxic effects of ayahuasca in rats, presenting data on neurodegeneration, while Jiang et al. (2015) suggested that the combined use of DMT and MAOI harmaline excessively activates the serotonergic system, inducing serotonin toxicity. Both 5HT2A and 5HT2C receptors play the major role in mediating DMT action (Ray, 2010). However, 5HT1A receptors have been related to the behavioral alterations caused by this drug, such as hypoactivity (Krebs-Thomson et al., 2006). Studies with mice found that administration of 5-Meo-DMT and harmaline reduce locomotion, but the effect is abolished by the administration of 5HT1A antagonists (Krebs-Thomson et al., 2006; Jiang et al., 2016). Moreover, serotonin and mainly dopamine can cross-regulate acetylcholine (Jackson et al., 1988; DeBoer et al., 1996). The decrease in acetylcholine levels due to increased serotonin and dopamine may also attributed to the lethargy observed after ayahuasca exposure.

The reduced exploration behavior observed in zebrafish exposed to 1 and 3 ml/L of ayahuasca was also associated with other behavioral parameters related to fear/anxiety. It is known that anxiogenic agents generally increase anxiety-like behaviors (Bencan et al., 2009; Egan et al., 2009; Maximino et al., 2010; Mathur and Guo, 2011; Pittman and Lott, 2014), which was corroborated by our data. Zebrafish exposed to 1 and 3 ml/L of ayahuasca exhibited high freezing/immobility and bottom dwelling behaviors (**Figures 1C, 2C**). Freezing is a complete absence of movements (except gills and eyes) that usually takes place at the bottom of the tank (Egan et al., 2009; Blaser et al., 2010; Kalueff et al., 2013). According to Elegante et al. (2010), Maximino et al. (2010), Oliveira et al. (2011), and Kalueff et al. (2013), freezing while at the bottom of the tank is usually the result of high stress/anxiety, which can be caused by exposure to toxic or sedative substances, when hypoactivity is also present. Thus, it seems that high concentrations of ayahuasca are responsible for increased anxiety-like behavior in zebrafish and future studies are needed to better understand how the brew produces this response. Furthermore, since co-administration of DMT and MAOI causes increased serotonergic activity, and harmaline also binds to many neurotransmitter receptors (norepinephrine, dopamine, glutamate receptors; Airaksinen et al., 1987; Miralles et al., 2005; Paterson et al., 2009; Iseri et al., 2011; Ossowska et al., 2014), it is important to investigate the extent to which ayahuasca use can be detrimental to animal behavior, physiology, and cognition.

In contrast to all the counterproductive effects of high ayahuasca concentrations, it seems that low concentrations act as an anxiolytic agent, as observed for the decreased bottom dwelling exhibited by zebrafish exposed to 0.1 ml/L (**Figures 1D, 2D**). This concentration did not affect locomotion (measured by speed and distance traveled) or freezing behavior, but clearly reduced bottom dwelling. Geotaxis behavior (also diving or bottom dwelling) is a very sensitive measure of anxiety characterized by fast and directed swimming toward the bottom (Kalueff et al., 2013). Bottom dwelling is commonly reduced by anxiolytic treatments (Bencan et al., 2009; Egan et al., 2009). Many anxiolytic drugs act by binding serotonin receptors (agonists) or by increasing serotonin availability [selective serotonin reuptake inhibitor (SSRI)], which agrees with the effects caused by ayahuasca. For instance, anxiolytic drugs such as fluoxetine and buspirone reduce freezing and bottom dwelling in zebrafish (Bencan et al., 2009; Egan et al., 2009). DMT is also a serotonergic agonist, which, in small amounts, seems to produce anxiolytic effects. Some studies have suggested that low concentrations of DMT decrease anxiety by binding trace amine receptors, thereby acting as a monoaminergic modulator (Jacob and Presti, 2005). The anxiolytic and antidepressant effects of ayahuasca are also suggested to be related to the β-carbolines harman, norharman and harmine, which were shown to reduce immobility and other anxiety-like behaviors in rodents subjected to forced swimming and high cross-maze tests (Aricioglu and Altunbas, 2003; Farzin and Mansouri, 2006). However, according to a number of studies, DMT combined with MAOIs such as harmaline can induce profound hallucinogenic effects (Ott, 2001; Halberstadt, 2016) and the use of this combination deserves further research to elucidate its toxicological impact.

Overall, our results emphasize that the zebrafish is an accurate, reliable, and profitable animal model for throughput screening and basic translational research of psychoactive drugs. The use of ayahuasca has been increasing worldwide and there is no regulatory legislation or toxicological studies stipulating a minimum consumption age, maximum recommended concentration to avoid deleterious effects or the consequences of chronic use. As such, doses and usage frequency should be considered because harmful effects are caused by high concentrations. We observed that very low concentrations positively affected zebrafish, but additional studies are needed for a thorough understanding of ayahuasca’s effects, since it seems to have potential as an alternative anxiolytic drug.

## Author Contributions

AL has designed the methodology; RS, DP, and JP-d-S have collected data; FF and AL have analyzed data; PS and AL have written the article; MY has quantified ayahuasca alkaloids; and BL-S has acquired ayahuasca and revised the article.

## Conflict of Interest Statement

The authors declare that the research was conducted in the absence of any commercial or financial relationships that could be construed as a potential conflict of interest.
